# An Open-Source Implementation of the Scaffold Identification and Naming System (SCINS) and Example Applications

**DOI:** 10.1021/acs.jcim.4c01314

**Published:** 2024-10-15

**Authors:** Kamen P. Petrov, Andreas Bender

**Affiliations:** † Pangea Bio, Pangea Botanica GmbH, Hardenbergstrasse 32, 10623 Berlin, Germany; ‡ Centre for Molecular Informatics, Yusuf Hamied Department of Chemistry, University of Cambridge, Lensfield Rd, CB2 1EW Cambridge, United Kingdom

## Abstract

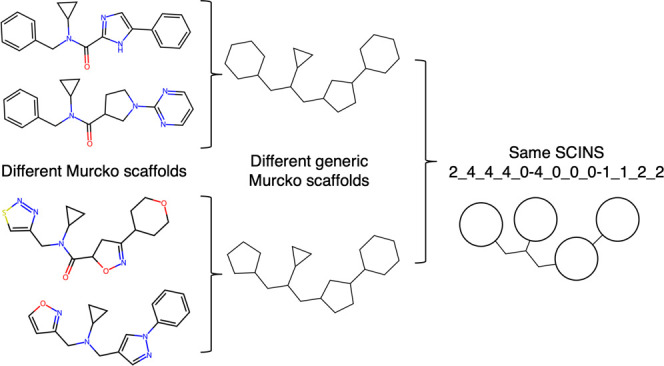

Organizing and partitioning sets of chemical structures is of considerable practical significance, e.g., in compound library analysis and the postprocessing of screening hit lists. Approaches such as unsupervised clustering are computationally demanding and dataset-dependent; on the other hand, rule-based methods, such as those based on Murcko scaffolds, have linear time complexity but are often too fine-grained, leading to a large number of singletons or sparsely populated classes. An alternative rule-based method that seeks to achieve an optimal balance when grouping compounds into sets is the ‘Scaffold Identification and Naming System’ (SCINS). To facilitate public use of this previously published method, here, we provide an open-source Python implementation of SCINS, dependent only on RDKit. We show that SCINS can be useful in identifying sparsely and densely populated regions in chemical space in large databases, here exemplified with Enamine REAL Diverse and ChEMBL. We find that Enamine REAL Diverse covers a much smaller SCINS space relative to ChEMBL, whereas the opposite is true when Murcko and generic Murcko scaffolds are considered. Additionally, we show that SCINS can result in chemically intuitive grouping of medium-sized sets of bioactive compounds, which can be useful in compound selection from virtual screening campaigns as well as postprocessing of experimental hit lists. Hence, in this work, we provide both an open-source implementation of SCINS and its characterization with relevant use cases.

## Introduction

Chemical space^1^ can be defined as the set of all stable small molecules under some practical constraints considering size, composition. Estimates of the size of drug-like chemical space range to about 10^60^ for organic molecules of up to 30 heavy atoms,^2^ and the space is for all practical purposes infinite. In practice only small subsets of chemical space are explored for a variety of applications in drug discovery. Hence, partitioning chemical data sets can enable researchers to focus on specific regions of chemical space, providing insights into the underlying structure and accelerating drug discovery by targeting molecules with desirable properties.

Over the years researchers have developed a large number of methodologies for subsetting chemical data sets.^3,4^ Two of the most common ones are clustering and rule-based methods.

At the root of many clustering algorithms lies the notion of similarity or distance between molecules because such algorithms aim to maximize intracluster similarity of member compounds and minimize similarity to other clusters. In order to compute the similarity between compounds, these unsupervised learning techniques most often rely on a vectorized descriptor to represent a molecule, normally a binary fingerprint.^5,6^ However, nonlinear molecular representations such as Feature Trees,^7^ where groups of atoms are represented by a single node in a tree, have also been introduced with similarity between trees being computed based on matching subtrees between two feature trees. Tanimoto and Cosine coefficients are some of the most popular choices for computing similarity between compounds based on binary fingerprints.^8^ For example, sequential agglomerative hierarchical non-overlapping (SAHN) algorithms, such as Ward’s clustering^9^ or unweighted pair group method with arithmetic mean (UPGMA),^10^ start with all compounds as singleton clusters and iteratively merge the most similar ones according to a distance or, alternatively, similarity criterion.^11^ The obtained clustering result could be used for diverse and representative subset selection,^12,13^ chemical series identification^14^ or structure–activity relationship (SAR) analyses.^15^ It is worth noting that these methods are often more computationally expensive relative to rule-based methods, and are also dataset-dependent, meaning that upon changing the dataset, the clustering results might change drastically.^3^

**Figure 1:**
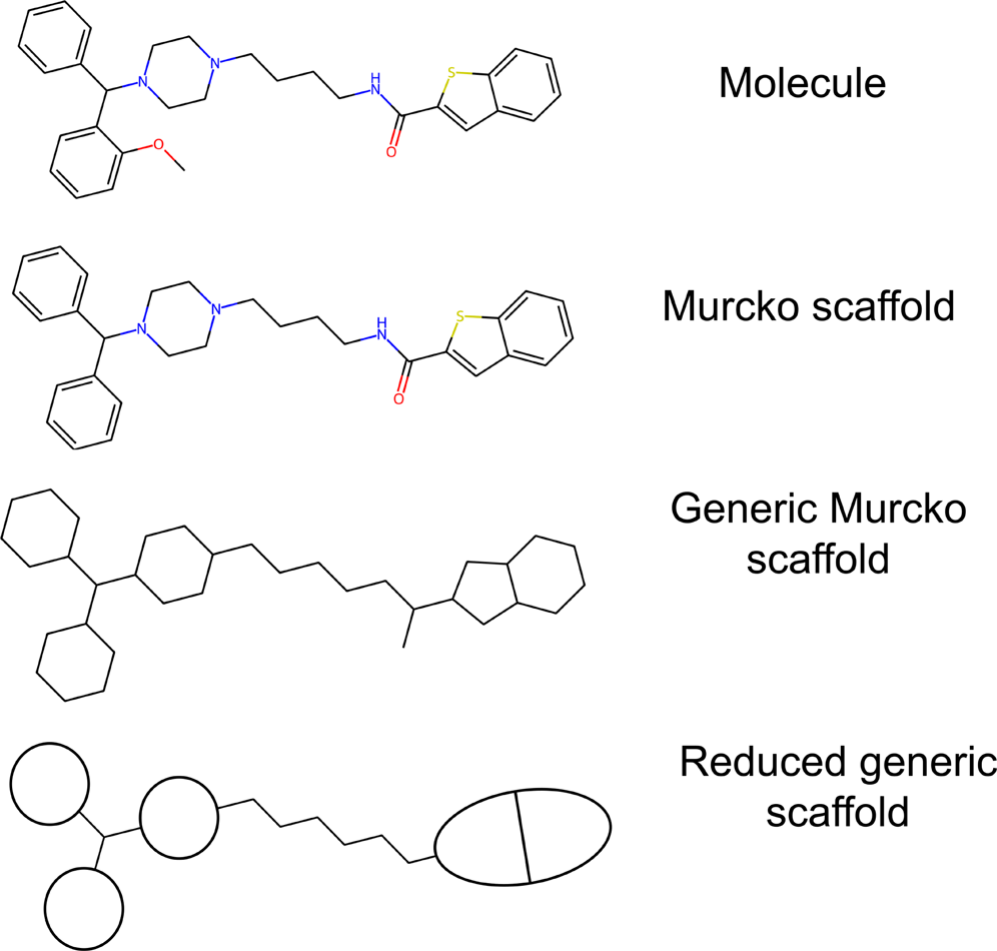
Illustration of some of the different ways to abstract a molecule and its scaffold. Murcko scaffolds are obtained from the molecule by removing the side chains. Generic Murcko scaffolds ignore bond and atom-type information. Reduced generic scaffolds (as described by SCINS) ignore ring size information and connectivity.

In contrast, the usefulness of rule-based methods lies in their interpretability, linear scaling with dataset size, and dataset-independence (meaning that the result of a class assignment for a given compound is independent from the overall composition of the dataset).^3,16^ As the name suggests, rule-based algorithms rely on the chemical structure and apply rules to determine the class of a given compound. For example, compounds can be grouped together based on the commonality of their molecular frameworks, a concept introduced by Bemis and Murcko.^17^ The molecular frameworks, also known as Murcko scaffolds, are defined as the part of the molecule that contains all ring systems and chains that link them, while all side chains are removed. Bemis and Murcko also described the graph framework obtained by disregarding atom and bond types, which later became known as the generic Murcko scaffold,^17^ as shown in Figure 1. The reason for abstracting the structure further is that changing atom and bond types results in a different scaffold, and thus compound class. Xu and Johnson^18^ explored how different levels of abstraction can create a hierarchical classification, where the leaves are the molecular frameworks, and the root, the most abstract molecular representation, is the reduced-skeletal cyclic system (similar to the reduced generic scaffold shown in Figure 1), where ring size information is disregarded and for which a molecular equivalence index (MEQI) descriptor was invented.^19^ On the other hand, Schuffenhauer et al.^20^ explored a different strategy for creating a hierarchy - the Scaffold Tree. The central idea is to sequentially remove parts of the Murcko scaffold based on predefined rules and group compounds together at the different levels of the tree. Additionally, more abstract representations of the Murcko scaffold for nonhierarchical grouping of structures have also been developed. For example, Scaffold Keys^21^ are a set of 32 simple topological and structural descriptors (keys) that are calculated on the basis of Murcko scaffolds. Another abstracted scaffold descriptor developed specifically for the analysis of DNA-encoded libraries (DELs), where the information at which position the DNA tag is attached to a molecule is important, is the reduced complexity molecular framework (RCMF) descriptor.^16^ Rings are grouped into predefined ring classes, chain linkers are described by their length, omitting atom-type information, and the connectivity between rings is described by the topological angle at which they are linked.

After their introduction in the 1990s, Murcko scaffolds became popular for analyzing and comparing chemical databases.^17,21−23^ For example, in their work^17^ Bemis and Murcko analyzed a set of around 5000 drugs and found that ca. 25% of these drugs were represented by the 42 most frequently occurring Murcko scaffolds and ca. 50% by the 32 most frequent generic Murcko scaffolds. Moreover, Lipkus et al.^25^ found that half of the CAS registry, containing more than^24^ million organic compounds at the time, could be described by only 143 generic Murcko scaffolds. Recently, Buehler and Reymond^26^ conducted a scaffold analysis of GDB-13^27^ and compared it to public databases of known compounds such as ZINC^28^ and PubChem.^29^ The authors concluded that while Murcko scaffolds in public databases mostly contained linker bonds and six-membered rings, the Murcko scaffolds in GDB-13 had diverse ring sizes and ring systems without linker bonds. The scaffold tree was used for visual representation purposes in the Scaffold Hunter software,^30^ and in the compound screening selection protocols to ensure coverage of available chemotypes in the process of the discovery of a potent RAF Inhibitor.^31^ RCMF^17^ was used to analyze and compare DELs, containing 10^8^–10^12^ compounds, in terms of their diversity by taking into account the number of compounds and Murcko scaffolds per RCMF class. The authors also showed the projection of chemical libraries onto a 2D RCMF map as a way to identify sparsely and densely populated parts of chemical space in a DEL.

**Figure 2:**
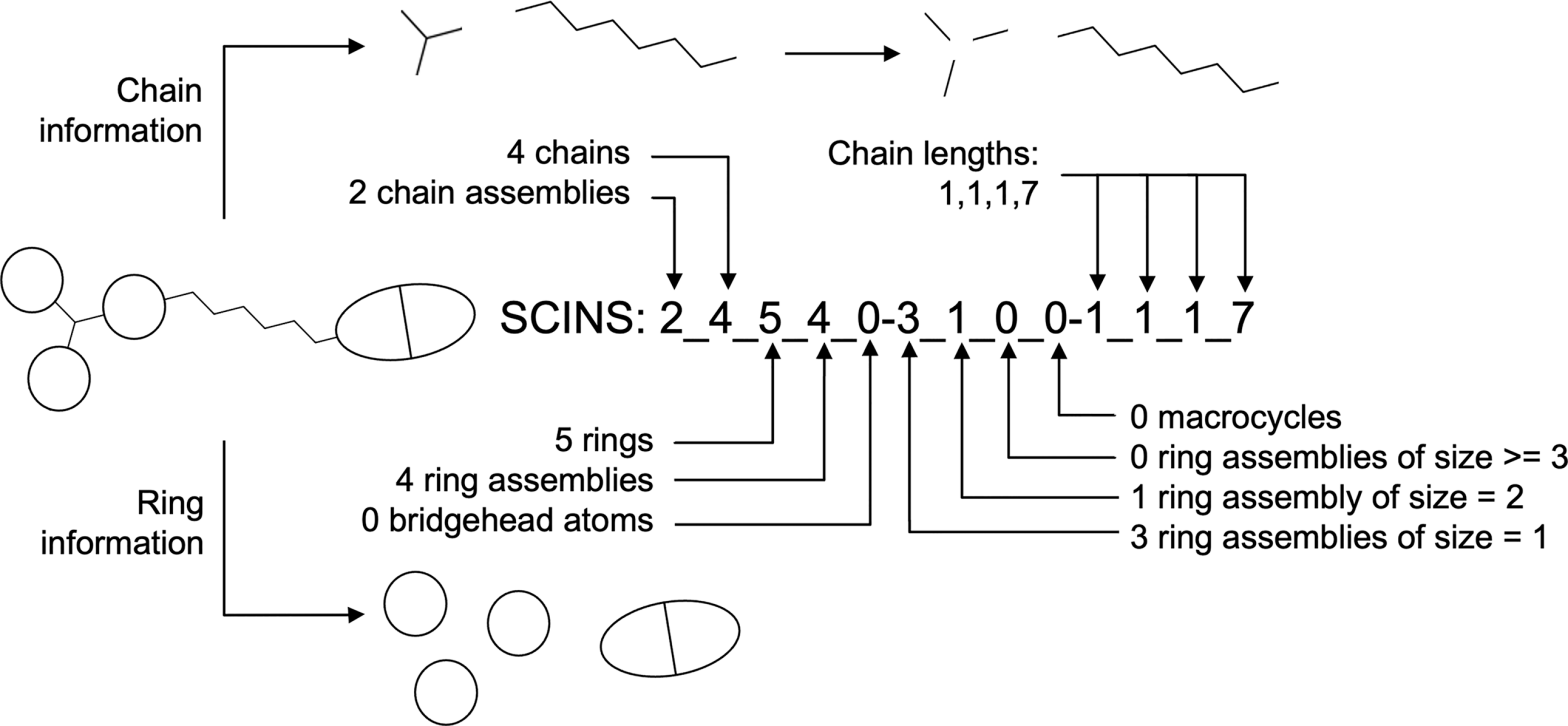
Illustration of the SCINS descriptor for the example molecule in Figure 1. SCINS consists of 13 numbers that describe the rings and chains in a reduced generic scaffold.

However, none of the above methods is “ideal” (if such a thing exists) in terms of being able to group chemical structures in a meaningful way; and different methods represent different trade-offs. With the aim to avoid the large number of singletons and small classes observed with Murcko scaffolds, as well as the unfavorable scaling of many clustering methods, the main focus of the current study is the Scaffold Identification and Naming System (SCINS).^32^ SCINS is a descriptor of the reduced generic scaffold (Figure 1), which is a further abstraction of the generic Murcko scaffold obtained by disregarding ring size, some chain length information and the topological connectivity of the generic scaffold. Figure 2 illustrates the different terms in the SCINS descriptor and how each of them is obtained for the example in Figure 1. SCINS was originally applied to small sets of structures with the aim to group together compounds of similar biological profiles (activity and safety pharmacology).^32^ In addition to providing an open-source implementation for general use, we apply SCINS to the analysis and comparison of two large databases: ChEMBL v33^33^ and the Enamine REAL (Readily Accessible) Diverse subset^34^ in SCINS, Murcko and generic Murcko scaffold space. We examine the distribution of populations of the different SCINS classes to gain insights into their diversity. Additionally, we describe the strengths and weaknesses of the SCINS approach to classifying chemical space by a case study on a compound set tested against the dopamine receptor D2 (DRD2) obtained from ChEMBL.^33^ Since SCINS has not been made available for public use before, we open-source our Python implementation which is dependent only on the RDKit toolkit.^35^

## Methods

### Data Curation

We chose ChEMBL v33^33^ (containing 1.9 million compounds labeled as a “Small molecule”) and the Enamine REAL Diverse subset^34^ (containing 48.2 million compounds) in order to assess the time necessary for classifying large databases and to compare the distribution of SCINS and scaffold classes between the two. The compounds in the diverse subset of Enamine REAL comply with Lipinski’s rule of five (Ro5)^42^ and Veber^43^ criteria: MW ≤ 500, SlogP ≤ 5, HBA ≤ 10, HBD ≤ 5, RotBonds ≤10, and TPSA ≤ 140, and have been filtered for PAINS^44^ and toxicophore substructures (as provided by the vendor). Furthermore, we obtained the compounds with a pChEMBL value of 5 or more and a standard relation annotation “=” that have been tested against human dopamine receptor D2 (DRD2) from ChEMBL v33^33^ in order to benchmark SCINS against Ward’s clustering in grouping together compounds of similar bioactivity. The averaged pChEMBL value was used for compounds with multiple values. This resulted in a set of 5699 compounds.

A standardization procedure was applied to all small molecules in ChEMBL, removing organometallic compounds, keeping only the largest fragment from a molecule (using rdMolStandardize.FragmentParent), neutralizing charged molecules (with rdMolStandardize.Uncharger) and standardizing tautomers (as the latter can influence Murcko scaffolds; done via rdMolStandardize.CanonicalTautomer).

All cheminformatics analysis was carried out using RDKit^35^ version 2023.09.5, unless otherwise stated.

### Murcko and Generic Murcko Scaffolds

**Figure 3:**
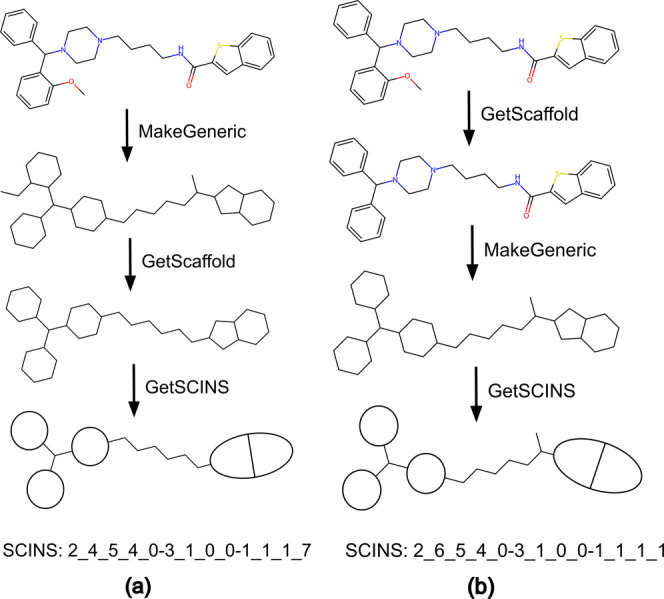
(a) Illustration of steps for obtaining SCINS as described in the current study for an example molecule. First, all atoms are converted to carbon and all bonds to single, resulting in the generic molecule. Second, the Murcko scaffold is obtained by striping all side chains, resulting in the generic Murcko scaffold of the molecule. (b) Illustration of a way to obtain the SCINS for the same molecule that follows the original definition of a generic Murcko scaffold.^17^ First, the Murcko scaffold is obtained by removing all side chains. Second, all atoms are converted to carbon and all bonds to single bonds, resulting in the generic scaffold. Finally, the SCINS descriptors are calculated, and the string is assembled. However, the scaffold has an extra chain which arises from the carbonyl oxygen. Therefore, the resulting Murcko scaffold, as well as the resulting SCINS, are different.

Two ways of obtaining the generic Murcko scaffold are illustrated in Figure 3. We opted for the method in Figure 3a which first converts all atoms to carbon and bonds to single bonds and then removes the side chains to obtain the generic scaffold. This results in a key difference to the method in Figure 3b - carbonyl similar groups do not affect the generic scaffold. The approach in Figure 3b, where first the scaffold is obtained and then generalized, was originally proposed by Bemis and Murcko because it would retain information about the presence and location of sp^2^ hybridized groups, such as the carbonyl. However, in the current case the generic Murcko scaffold is necessary to obtain SCINS which disregards topology. Therefore, we argue that an additional chain in the generic scaffold resulting from the carbonyl group would just add noise to the already very coarse SCINS descriptor. One caveat with our approach is that compounds containing atoms with valence higher than 4 (such as the sulfur in Aryl-SF_5_) would fail the first step (as this would result in a hexavalent carbon, which raises an error in RDKit upon sanitization). This could be avoided by changing the code that obtains the scaffold to also remove carbonyl and similar groups. We provide such a function in the SCINS package but do not use it in this work as in our experience it is slower by about 30% compared to the C++ implementation in RDKit.

### SCINS Descriptor

The Scaffold Identification and Naming System (SCINS)^32^ is a descriptor of the reduced generic scaffold (Figure 2) and is represented by a string in the format A_B_C_D_E-F_G_H_I-J_K_L_M where each letter is the value of one of the following 12 numerical descriptors:

**A. Number of Chain Assemblies.** Chain assemblies are contiguous linkers between ring assemblies. They are uncovered by removing all ring bonds in the molecule.

**B. Number of Chains.** Chains are all unbranched linkers needed to cover all nonring bonds in the molecule.

**C. Number of Rings.**

**D. Number of Ring Assemblies.** Ring assemblies are fragments remaining when all acyclic bonds have been removed.

**E. Number of Bridge Atoms**.

**F. Number of Ring Assemblies Consisting of Exactly One Ring.**

**G. Number of Ring Assemblies Consisting of Exactly Two Rings.**

**H. Number of Ring Assemblies Consisting of Three or More Rings.**

**I. Number of Macrocycles.** Rings constituting more than 12 atoms are considered macrocyclic.

**J. Binned Length of Shortest Chain.**

**K. Binned Length of Second Shortest Chain.**

**L. Binned Length of Third Shortest Chain.**

**M. Binned Length of Fourth Shortest Chain** (Table 1).

**Table 1:** Binning Rules for Chain Lengths

chain length	0 (no chain)	1	2	3, 4	5, 6	≥7
binned length	0	1	2	3	4	7

### Implementation

A pivotal part of the algorithm for computing the SCINS for a generic scaffold is obtaining all atoms that take part in a chain (equivalent to removing all ring atoms from the molecule). The so-obtained chains are then traversed in a depth-first type of manner and used to find the contiguous linkers (A), as well as computing the lengths of the chains in the chain assemblies (B, J-M). To calculate the ring descriptors, we first obtain a list of all rings (the length of which is C), where each ring is represented by a list of atom indices that take part in it, and then, merging rings with common atoms gives the ring assemblies. The result is then used to compute D and F–H. E is different from the original definition which uses the number of bridging bonds.^32^ Here the number of bridge atoms is used because of the convenience of the method implemented in RDKit (rdMolDescriptors.CalcNumBridgeheadAtoms). While users have the choice to obtain the generic scaffold in the way they choose, throughout the rest of this work we use the sequence in Figure 3a because it results in a more generic scaffold, therefore, reducing scaffold and SCINS space. Empirically, this resulted in a much smaller SCINS space (30–40% depending on the dataset).

### Implementation Details

The package is implemented in Python and relies only on the RDKit toolkit^35^ (v. 2023.09.5) as a dependency. The API gives access to the main function that calculates the SCINS from the generic Murcko scaffold of the molecule (generic_scaffold_mol_to_scins, generic_scaffold_smiles_to_scins), which gives the user the freedom to choose how to obtain the generic scaffold. Additionally, it also provides separate functions for individual terms in SCINS (mol_to_num_chain_assemblies, mol_to_chain_lengths, and mol_to_num_ring_assemblies). While SCINS can be obtained also by sequentially applying those functions in a pipeline, this would lead to calculating certain variables multiple times. The generic_scaffold_mol_to_scins function is optimized to avoid this.

In case of an error when calculating the SCINS, the package returns “ERROR_SCINS” which can easily be detected in the later parts of a workflow. In our experience this has only been observed when a rdkit.Chem.rdchem.Mol object (i.e., a molecule that can be sanitized according to the rules in RDKit) cannot be obtained from the input.

### Ward’s Clustering Benchmark

SCINS was benchmarked against Ward’s agglomerative hierarchical clustering^9^ (as implemented in SciPy^36^) in terms of grouping together compounds of similar bioactivity against DRD2. We computed the (1 - Tanimoto similarity) distance matrix from the Morgan fingerprints^6^ (radius = 2, size = 2048) in the DRD2 compound set using DataStructs.BulkTanimotoSimilarity(returnDistance = True). Subsequently, scipy.cluster.hierarchy.ward function and the distance threshold was set to 1.46, such that the number of clusters produced matches the number of categories resulting from the application of SCINS.

### Assessing the Utility of SCINS in a Clustering Setting

For the cluster analysis of the DRD2 ChEMBL set we used the weighted average class spread (SP).^34^
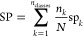
1Where *n_k_* is the size of the class *k*, *N* is the total number of compounds in the dataset, *n*_classes_ in the number of classes, and sp*_k_* is the class spread for class *k*, defined by
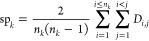
2where *i*, *j* is a pair of compounds within the same class and *D_i,j_*, in this work is defined as
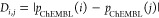
3where *p*_ChEMBL_(i) is the *p*_ChEMBL_ value for *i* in an assay against a single target in ChEMBL (in this case DRD2). This is different from the definition in the original SCINS paper, where the biological profile distance *D_i,j_* was defined on the basis of activity in multiple assays.

### Assessing SCINS and Scaffold Diversity

In order to quantify the diversity of the analyzed databases, we followed the procedure described in ref (37). We computed the scaffold retrieval curve (SRC) by first ordering the scaffolds or SCINS classes by their frequency of occurrence (most to least common). Additionally, the fraction of scaffolds or SCINS required to describe half the dataset was obtained and referred to as F_50_.

Furthermore, the scaled Shannon entropy (SSE) was obtained also according to ref (37). It represents a measure of the distribution of the compounds across the different classes and is, therefore, complementary to the curves described above in analyzing the diversity. First the Shannon entropy (SE) is defined as
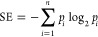
4where *n* is the number of classes (SCINS or scaffolds), *c_i_* is the number of compounds with that class, *P* is the total number of compounds (the population), hence *p_i_* is the relative frequency of the class in the dataset.
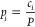
5The so-defined SE can range between 0 (all compounds are in a single class) to log_2_ *n* (all compounds are uniformly distributed across the different classes). The scaled Shannon entropy (SSE) is normalized for the different n values such that it ranges from 0 to 1.
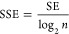
6

## Results

### Runtime Benchmarking

We first investigated the time it takes to classify ChEMBL and Enamine REAL Diverse using SCINS and benchmarked that against obtaining the Murcko and generic Murcko scaffolds. As the results in Table 2 show, SCINS is the slowest among the three, taking just under an hour and 2.5 hours to classify ChEMBL v33 and Enamine REAL diverse, respectively. This is to be expected since in order to obtain the SCINS for a molecule, first the generic Murcko scaffold has to be determined. Hence, the time needed for obtaining the SCINS from the generic Murcko scaffold is approximately the difference between the entries for SCINS and generic Murcko scaffolds in Table 2. As with the other rule-based methods, the computation of SCINS can easily be parallelized to decrease run times. Therefore, we conclude that SCINS is amenable to compound sets with tens of millions of members.

**Table 2:** Time Required to Classify the Two Databases with the Different Methods

	number of compounds	SCINS	generic Murcko scaffolds	Murcko scaffolds
ChEMBLv33^a^	2.4 M	53.5 min	29.3 min	15.0 min
Enamine REAL Diverse subset^b^	48.2 M	149.0 min	120.1 min	47.4 min

a Times shown for 1 core on an 8-core Intel Core i7–118G7 CPU at 3.0 GHz (local machine).

b Times shown for 6 cores on an 8-core AMD EPYC 7R13 CPU at 3.6 GHz (AWS EC2 c6a.2xlarge).

### Comparison of SCINS and Murcko Scaffolds on Enamine REAL Diverse and ChEMBL Libraries

Next, we analyzed the mapping behavior of molecular to SCINS space coverage for both libraries and compared that to the results from Murcko and generic Murcko scaffolds classification. The results of this analysis are shown in Table 3 and it can be seen that the 20-fold increase in the number of compounds from 1.9 million in ChEMBL to 48.2 million in Enamine Diverse results in a nearly 25-fold increase in the number of Murcko scaffolds (from 580,000 to 17.4 million, respectively), but less than a 2-fold increase in the number of generic Murcko scaffolds (from 130,000 to 240,000, respectively). Strikingly, the number of SCINS classes for Enamine Diverse is ∼300, or 25 times *lower* than the ∼7500 SCINS classes in ChEMBL, and about 600 times lower when put in relation to the number of compounds in each set. These observations could be explained by considering the chemistries in the two libraries. On the one hand, Enamine REAL is a synthesize-on-demand virtual library built by coupling in-stock building blocks with prevalidated reactivity using well-established parallel chemistry reactions.^3^ Hence, most compounds in the library are made using a small number of reactions (such as amide coupling, click reactions, etc.) by combining a variety of available building blocks.^38,39^ In agreement with previous research, this leads to superior scaffold diversity.^29,39^ However, the generic Murcko scaffolds and SCINS (Table 3) are a lot less diverse. While keeping the reaction type the same, a change in atom types in the building blocks with the same topological connectivity leads to a different Murcko scaffold, but the same generic Murcko scaffold and SCINS (Figure S1–Sn). Changing the size of a ring and/or the connectivity pattern between rings and chains further changes the generic Murcko scaffold, but the SCINS remains in most cases unchanged because it neglects topology and ring size (Figure S2–Sn). Therefore, the combinatorial element in the building strategy that underlies Enamine REAL is one likely reason for the larger number of Murcko and generic Murcko scaffolds per SCINS class, indicating that a more densely populated *subset* of chemical space in that library.

**Table 3:** Number of Unique Molecular Representations for the Two Databases Analyzed^a^

	number of compounds	number of SCINS	number of generic Murcko scaffolds	number of Murcko scaffolds
ChEMBL v33	1.9 M	7563	132,381	579,279
ChEMBL filtered to comply with Ro5 and Veber criteria	1.3 M	1520	63,785	394,043
Enamine REAL Diverse subset	48.2 M	314	239,100	17,423,904

a It can be seen that, on average, the SCINS classes in Enamine REAL are more densely populated than those in ChEMBL.

On the other hand, ChEMBL is not restricted by a defined set of reactions and building blocks, but rather covers literature-reported usually biologically active compounds. In contrast to Enamine REAL, this results in a much larger SCINS space covered. However, this is sparsely populated as indicated by the lower average number of compounds, Murcko and generic Murcko scaffolds per SCINS class. This is further supported by Figures S3–Sn and S4–Sn, which show that the population of SCINS classes is highly skewed toward sparsely populated classes, unlike in the case of Enamine Diverse, where this distribution is relatively more even. For example, a small number of macrocyclic compounds (25,070) corresponds to a substantial number of SCINS classes (1609), whereas Enamine REAL Diverse contains no macrocycles. Hence, a significant portion of ChEMBL SCINS space is sparsely populated when considering the number of distinct structures per SCINS.

### Impact of Molecular Property Distributions on SCINS and Murcko Diversity Obtained

We next computed the molecular weight distributions of the compounds for each database in order to understand whether those might be influencing the SCINS classes distributions. The results are shown in Figures S5–Sn and S6–Sn, where it can be seen that while the molecular weight distribution for ChEMBL has a long tail of large molecular weights, the one for Enamine REAL Diverse fits between 96 and 507 Da without such a tail. Filtering out compounds outside of this molecular weight range in ChEMBL, as well as the additional Ro5^42^ and Veber^43^ criteria with which the compounds in Enamine REAL Diverse comply (as detailed in Methods), results in the loss of around 600,000 compounds (or about 30% of the dataset, leading to 1.3 M compounds) and the loss of 6000 SCINS classes (or about 80% of the total number of SCINS classes in ChEMBL). Hence, it can be seen that larger molecules contribute disproportionately to SCINS diversity in case of ChEMBL. This is to be expected as heavier compounds containing more heavy atoms can have a larger variety of rings and chains. The distribution of populations in SCINS classes of the resulting ChEMBL set is shown in the box plot in Figure S7–Sn and it is noticeably less skewed. Hence, the majority of SCINS classes in ChEMBL arise from a minority of members with properties outside of the ranges in Lipinski’s Ro5 and Veber’s criteria. However, the number of SCINS classes in ChEMBL after the application of the filters is around 1500, still significantly higher than that in Enamine REAL Diverse, highlighting the constraints implied in combining building blocks to obtain Enamine REAL space.

On the other hand, filtering out the same compounds from the ChEMBL set has a weaker effect on the Murcko and generic Murcko scaffold counts, decreasing by about 32% (nearly as much as the molecules) and about 50%, respectively, as can be seen in Table 3. Hence, the effect of skewing class population distribution by molecules outside the Lipinski’s Ro5 and Veber’s criteria decreases as with the amount of information captured by the scaffold descriptor. Thus, if SCINS were to take into account ring sizes, probably a smaller proportion of the SCINS classes would account for the molecules outside the previously mentioned property filters.

### Class Population Analysis of Enamine REAL Diverse and ChEMBL Using SCINS

**Figure 4:**
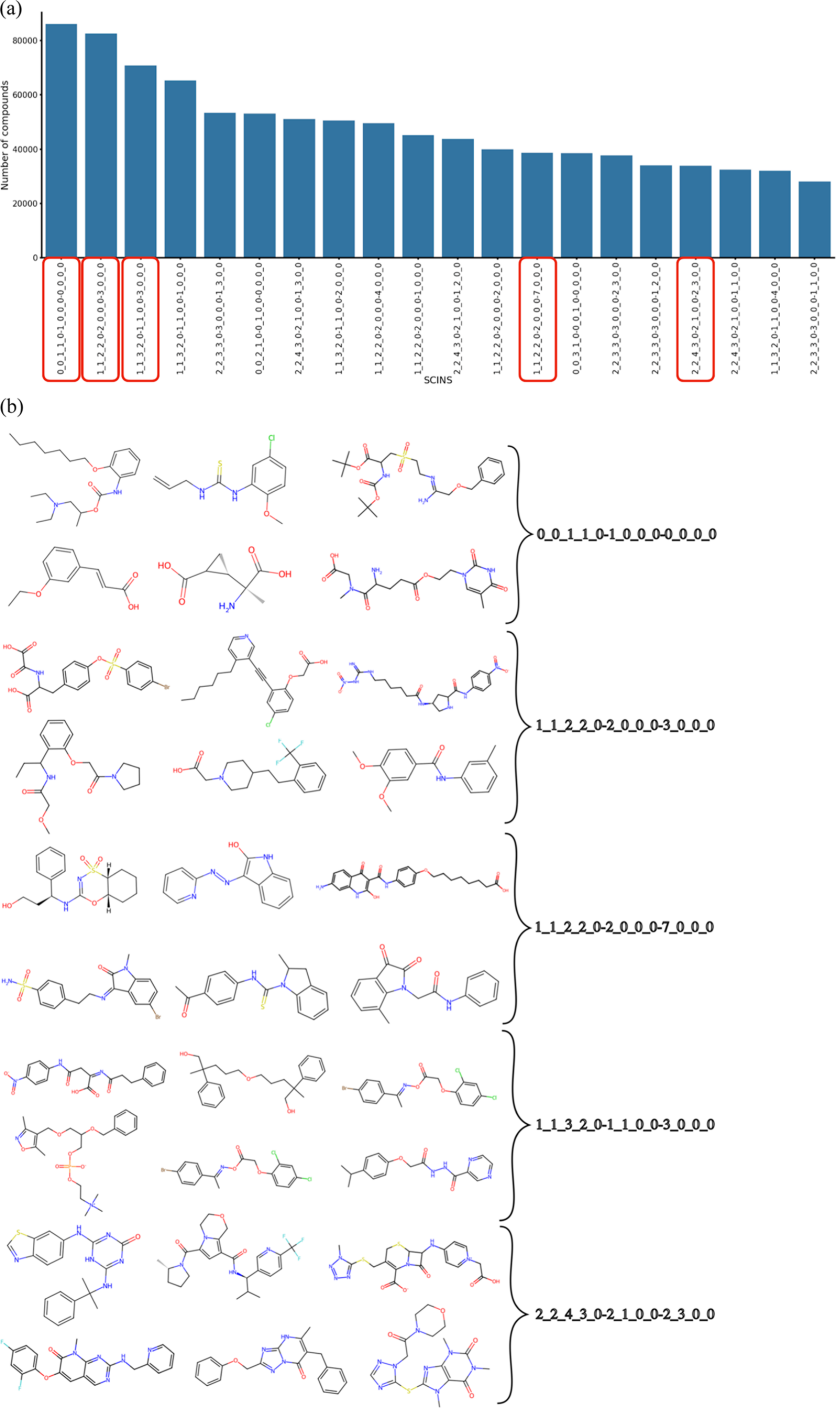
(a) Top 20 most populated SCINS classes for ChEMBL. The highlighted classes are also some of the most populated ones in Enamine REAL Diverse (see Figure 5). (b) Examples of ChEMBL compounds from the highlighted classes in (a).

**Figure 5:**
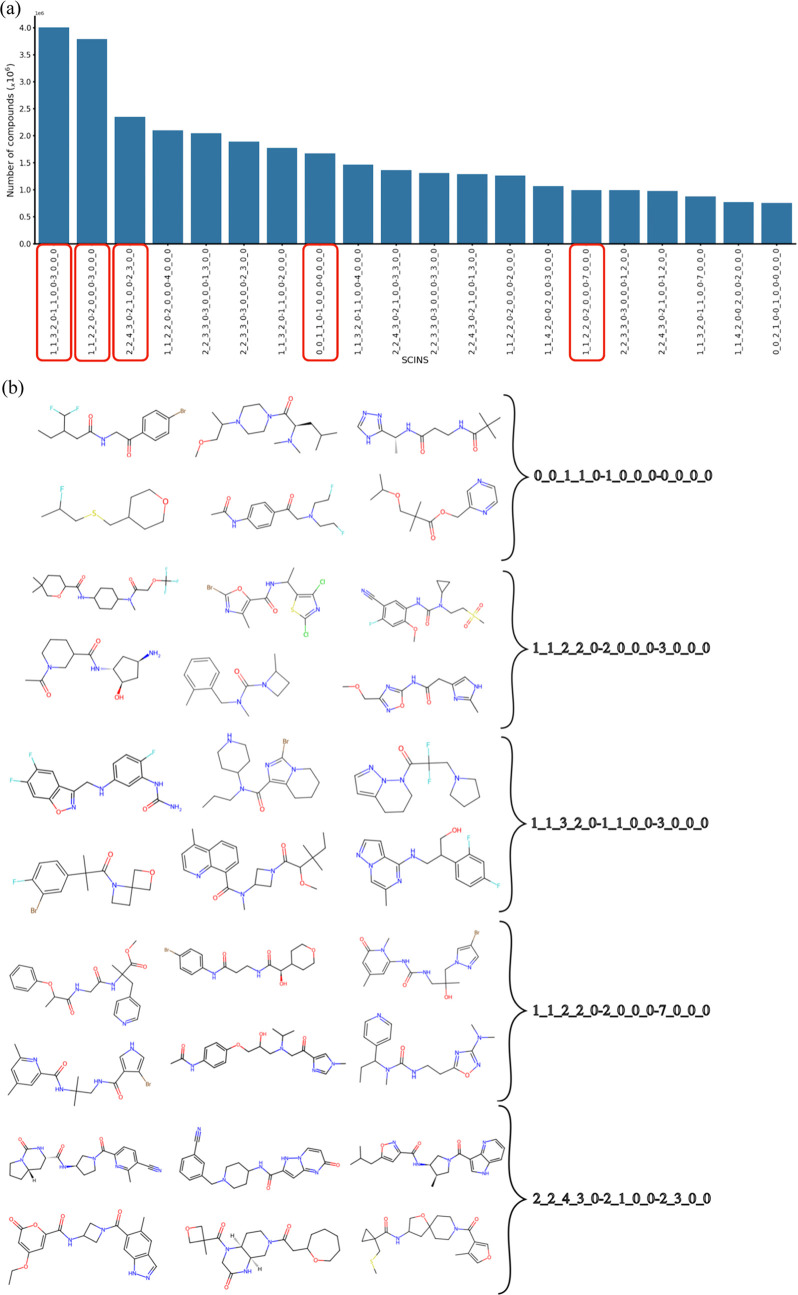
(a) Top 20 most populated SCINS classes for Enamine REAL Diverse. The highlighted classes are also some of the most populated ones in ChEMBL (see Figure 4). (b) Examples of Enamine REAL Diverse compounds from the highlighted classes in (a).

We next investigated the differences in population of the 20 most populated SCINS classes for the two databases. This is shown in Figures 4a and 5a where it can be seen that 15 of the SCINS classes are common across the two plots, despite not being in the exact same order. Additionally, Figures 4b and 5b show 10 random molecules for 5 of the common SCINS classes as examples. While it is difficult to judge the differences in chemistry from these examples, the long aliphatic chains in the ChEMBL examples are reminiscent of natural products, while most of the compounds in Enamine REAL Diverse would be typically synthetic. Hence, the commonality in SCINS classes does not imply chemical similarity, which is to be expected since SCINS ignores most of the chemical information. Moreover, nearly 88.3% of Enamine Diverse molecules have 2–4 rings, 2–3 ring assemblies, and 1–2 chain assemblies, while this range covers only about 59.7% of ChEMBL. Hence, Enamine REAL Diverse covers this particular part of SCINS space especially densely. This result is similar to the results obtained in the scaffold analysis of the CAS database, where the authors found that half of the compounds can be described by only 143 framework shapes.^26^ It is also similar to the analysis that Bemis and Murcko conducted with the introduction of the molecular frameworks, which showed that more than half of the known drugs at that time were described by only 32 scaffolds.^17^

### Database Retrieval Analysis of ChEMBL and Enamine REAL Diverse Using SCINS

**Figure 6:**
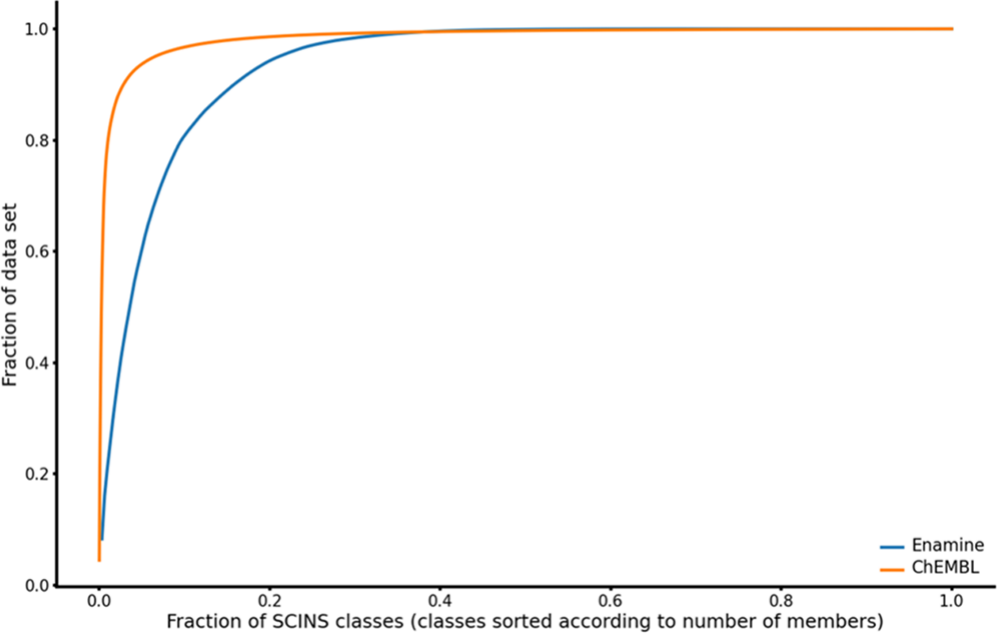
SCINS retrieval curves for Enamine REAL Diverse and ChEMBL.

In order to quantitatively assess the diversity of ChEMBL and Enamine REAL Diverse we next calculated the SCINS retrieval curves (SRCs) for the two databases, shown in Figure 6. While both curves are far from the diagonal, indicating a heavy-tailed distribution (as also seen in Figures S3–Sn and S4–Sn), the one for Enamine encloses a smaller AUC (0.939, while that for ChEMBL is 0.985). Additionally, 50% of the dataset is contained in only 0.2% of the SCINS classes in ChEMBL but 3.8% in Enamine. The scaled Shannon entropy (SSE) also suggests that compounds in the top N most populated SCINS classes are more equally distributed across those N classes in the case of Enamine Diverse compared to ChEMBL, as shown in Table 4. Hence, the three observations agree and confirm the more even distribution of molecules across the SCINS classes for Enamine REAL Diverse compared to ChEMBL. However, when the absolute number of SCINS classes is considered on the *x*-axis, Enamine REAL Diverse expectedly plateaus first, as shown in Figure S8–Sn.

**Table 4:** SCINS Retrieval Analysis

	AUC	F_50_	SSE top 10	SSE top 50	SSE top 100
Enamine REAL diverse	0.939	3.8%	0.607	0.839	0.828
ChEMBL	0.985	0.3%	0.472	0.747	0.766

### Application of SCINS to the DRD2 Set from ChEMBL

We next examined the ability of SCINS to group compounds of the same bioactivity together by applying SCINS to the DRD2 set and compared that to the result from classification by Murcko scaffolds, Ward’s clustering and random classification, as explained in Methods. The results are shown in Table 5 and it can be seen that Murcko scaffolds resulted in the lowest weighted average class spread of 0.37 (SP, eq 1), but also the highest number of singletons (almost 2000 out of the 5699 compounds in the set, or more than a third). This is to be expected as according to the SP eq (eq 1) a solution with only singletons would have an SP of 0 (which is the lowest possible value for SP). However, this is not practically useful as the number of classes is very large. Conversely, despite leading to a larger number of singletons than Ward’s clustering (90 and 0, respectively), the application of SCINS also resulted in a higher SP. This is in agreement with the findings presented in the original SCINS paper,^32^ where methods such as OptiSim^40^ on FCPF_4 fingerprints also performed better than SCINS in terms of lower class spread and number of clusters. One possible explanation for this result is that sets of bioactive compounds for a target tend to contain series of analogues with the same or similar substructures which can be identified by the fingerprint-based clustering leading to compounds of similar bioactivity in the same cluster due to a certain substructure. Finally, random classification expectedly led to the highest class spread of and no singletons. Hence, the results suggest that Ward’s clustering might be more suitable than SCINS when the aim is grouping compounds of similar activity together based on the DRD2 compound set.

**Table 5:** Comparison of the Classification Results from the Application of Murcko Scaffolds, SCINS, and Ward’s Clustering to the DRD2 Compound Set

	Murcko scaffolds	SCINS	Ward’s clustering	random
number of classes/clusters	2727	281	281	281
number of singletons	1940	90	0	0
SP (class spread, eq 1) (↓)	0.37	0.89	0.70	1.10

### Illustration of Some Members of a SCINS Class and Comparison to Ward’s Clustering Results

**Figure 7:**
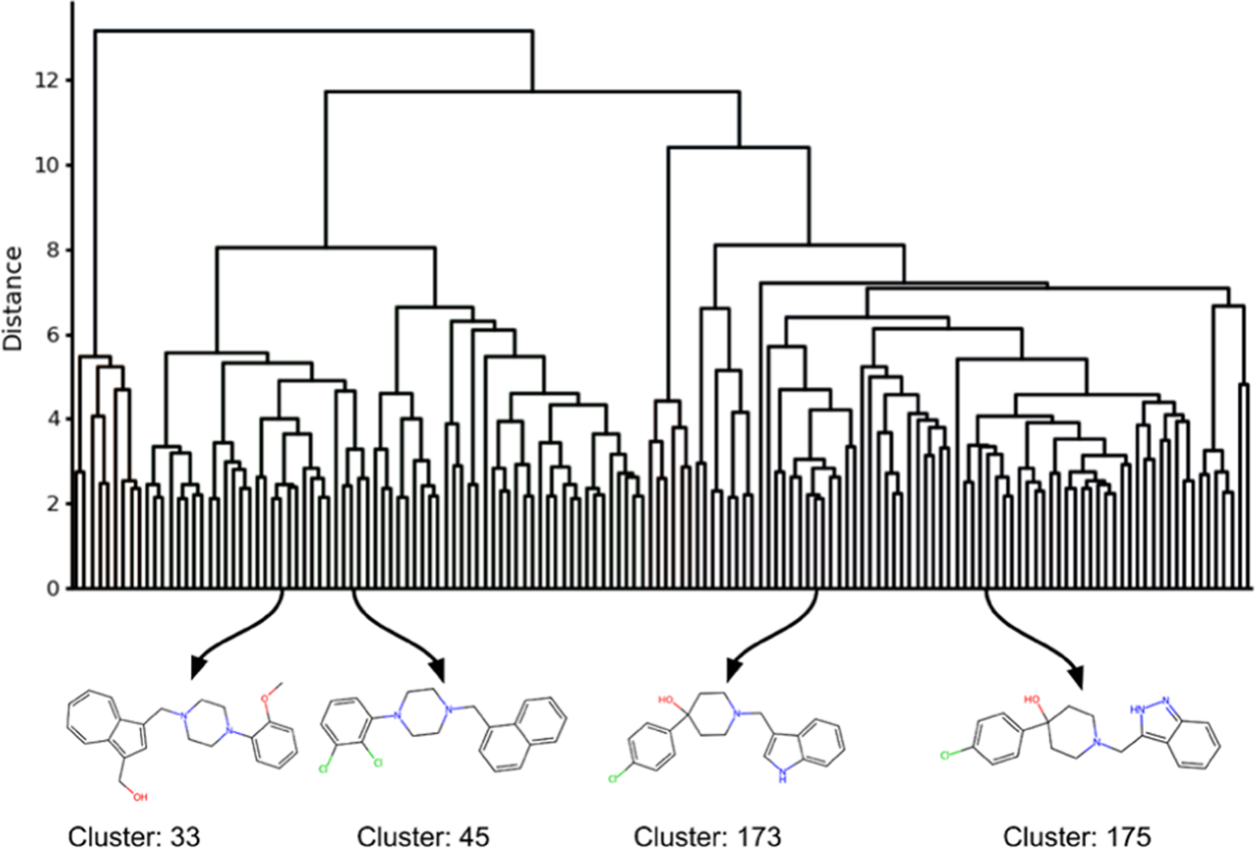
Compounds from the same SCINS class (2_2_4_3_0–2_1_0_0–1_2_0_0) constitute different Ward’s clusters, because of insufficiently high similarity in fingerprint space, despite the obvious “chemically intuitive” similarity. This is an illustration and not the exact dendrogram that the clustering resulted in, though the cluster indices and members are.

We next aimed to gain further insight into the chemical groupings obtained from SCINS compared to other methods on the DRD2 set. This is shown in Figure 7 and it can be seen that compounds have similar topologies and are described by the same SCINS (in this case 2_2_4_3_0–2_1_0_0–1_2_0_0), in spite of the difference in functional groups and pharmacophoric features. It is also worth noting that despite the SCINS descriptor being the same across the four examples, it comprises four different Murcko scaffolds, and three different generic Murcko scaffolds, further illustrating the higher level of abstraction achieved by SCINS. However, those molecules were put in different Ward’s clusters due to the lack of sufficient substructural commonalities at the distance threshold chosen to match the number of SCINS classes (corresponding to a Ward’s distance of 1.46). Therefore, in the context of the DRD2 set analyzed in this section, we can conclude that SCINS can provide a chemically intuitive way to partition compound sets.

**Figure 8:**
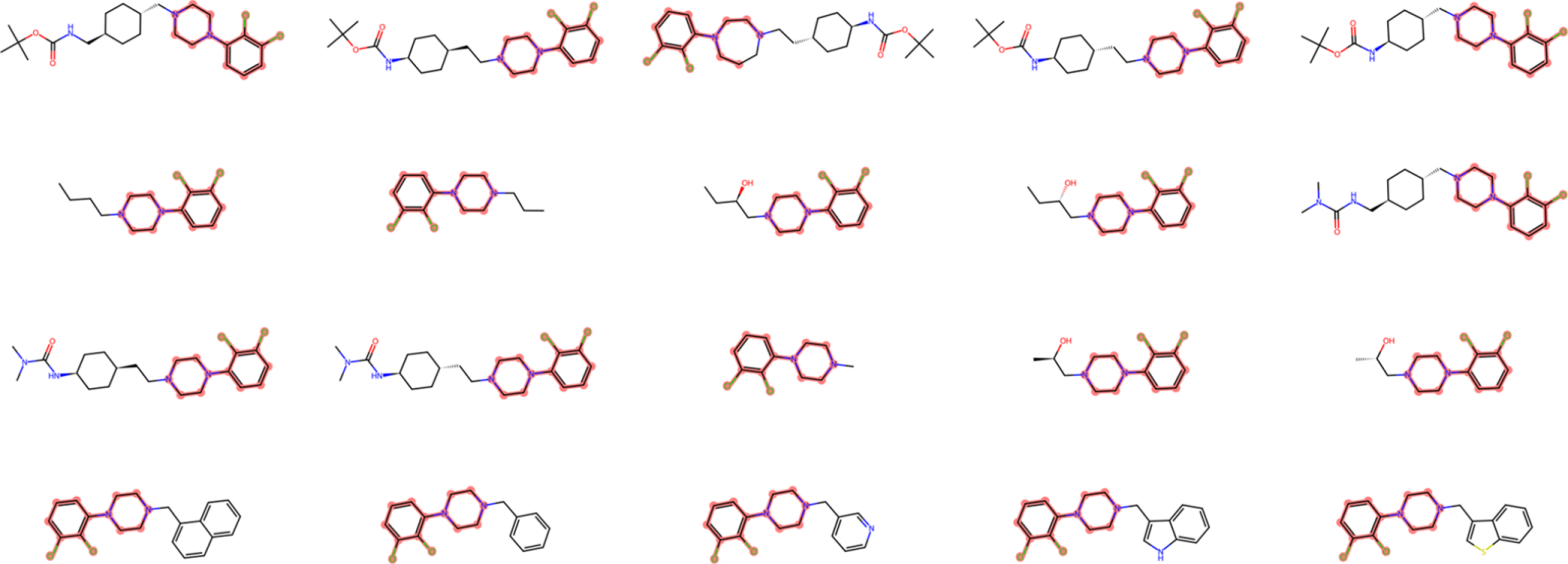
Some compounds from hierarchical cluster 45. The maximum common substructure (MCS) of those compounds is highlighted in red. It can be seen that compounds are generally dissimilar beyond the substructural similarity which rendered them a part of the same cluster.

Furthermore, we selected Ward’s cluster 45 from Figure 7 for visual analysis and illustrated its members in Figure 8, highlighting their maximum common substructure in red. It can be seen that the molecules contain a large common substructural motif but are also considerably different in size (molecular weight, number of heavy atoms, etc.) and even functionality. This may be desirable when the goal is chemical series identification (as Kruger et al. suggested^14^), but does not ensure that molecules are similar outside of the repeated structural motif. While Morgan fingerprints depend on the connectivity table (unlike SCINS), molecules with different functional groups are within the same cluster because the distance cutoff is relatively high for the set number of clusters that was used here. In order to ensure higher similarity of the compound clusters, one can decrease the distance threshold. However, this would increase the number of clusters substantially. Thus, we can conclude that Morgan fingerprint-based clustering tends to identify common substructures but does not ensure topological similarities beyond that substructure. This conclusion is also in agreement with the statement by Vogt^41^ that substructure-based fingerprints work best on small molecules comparable in size but do not adequately reflect the overall size or shape of a molecule well. However, it is worth noting that there are other fingerprints like atom-pair (AP)^45^ and MAP4^46^ which should be better able to deal with the differences in the sizes of molecules and overcome this limitation of Morgan fingerprints.

### Limitations of SCINS

**Figure 9:**
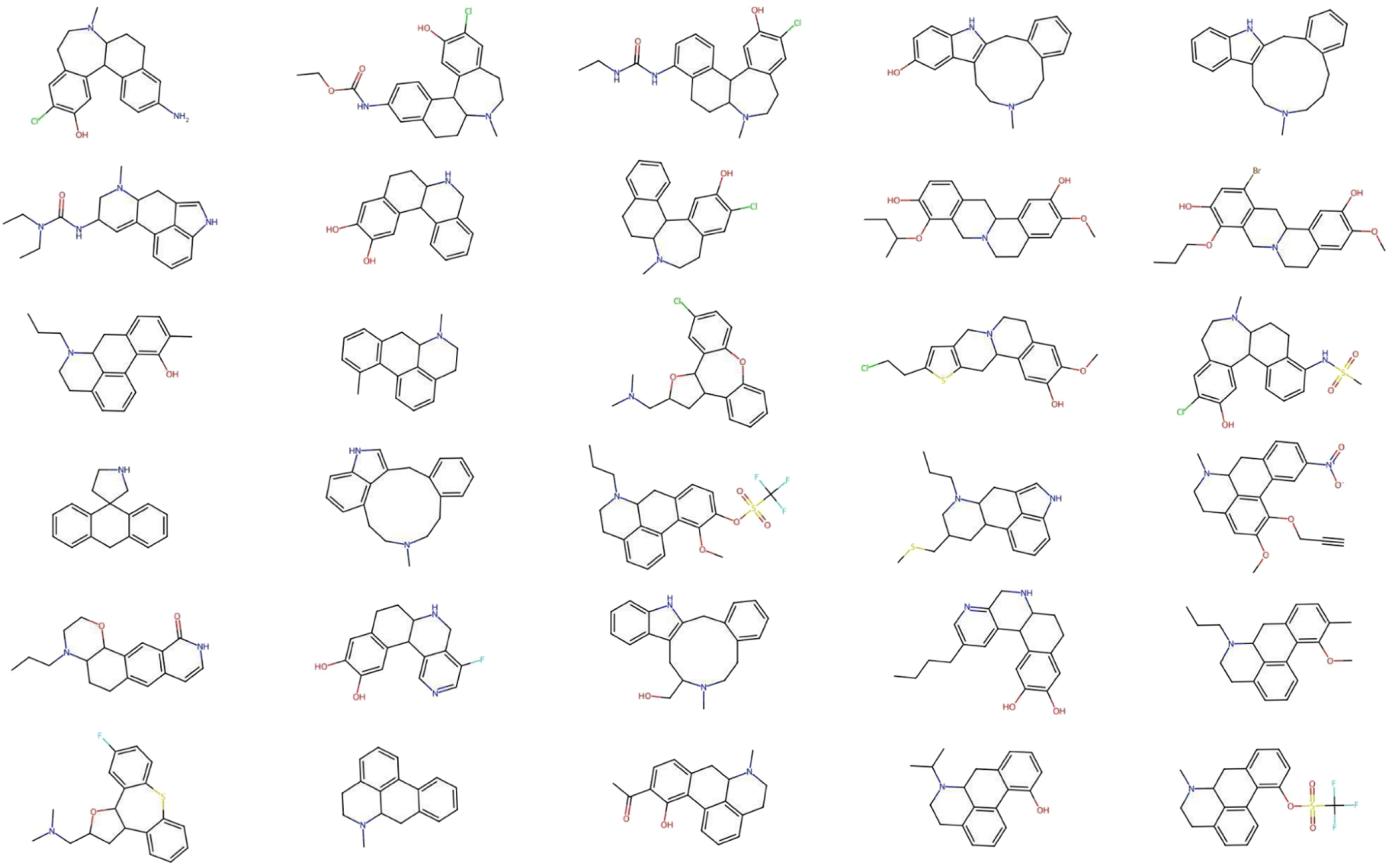
Compounds belonging to SCINS class 0_0_4_1_0–0_0_1_0–0_0_0_0. It can be seen that compounds in classes without chains in the scaffolds (as well as those without ring systems) can be very chemically dissimilar, due to the abstractions made upon obtaining the SCINS descriptor.

Similar to other scaffold-based approaches, SCINS does not lead to reasonable results for grouping compounds without any rings, since all of those compounds correspond to SCINS 0_0_0_0_0–0_0_0_0–0_0_0_0. Additionally, compounds with only a single ring assembly and no chains can also be highly dissimilar, depending on the dataset to which the method is applied. An example of this is illustrated in Figure 9 for compounds with SCINS 0_0_4_1_0–0_0_1_0–0_0_0_0, i.e., compounds with a single ring assembly consisting of 4 rings. Compounds are dissimilar, due to the insufficient chemical information that is captured by the different values used in the SCINS representation.

This further highlights the connectivity information loss which results from the abstractions made in SCINS. In Figure 9 this is the relative position of ring connectivity patterns between rings in fused ring assemblies, but it could also be the connectivity between rings and chains.

Finally, SCINS is also not aimed at classifying macrocyclic compounds even though one of the descriptors in the SCINS string is for the number of macrocycles in the compound. The authors recommend for macrocycles to be treated separately if any are identified after the application of SCINS to a dataset.

## Conclusions

Partitioning chemical data is a crucial part of drug discovery as it enables the better understanding and exploration of the practically infinite chemical space. In this work we introduced the open-source Python implementation of the rule-based method Scaffold Identification and Naming System (SCINS)^32^ and exemplified its application in the analysis and comparison of two large databases, namely ChEMBL^33^ and the Enamine REAL Diverse subset.^34^ We found that the full Enamine REAL Diverse subset was described by only ca. 300 SCINS classes, likely a result of the combinatorial synthesis strategy on which Enamine REAL is based and the compliance with Lipinski’s Ro5^42^ and the Veber criteria.^43^ On the other hand, even after applying the same physicochemical property filters to ChEMBL, the number of SCINS classes remained around 1500, suggesting a difference in the chemistry of bioactive compounds reported in literature that constitute ChEMBL from those in Enamine REAL. Additionally, the distributions of compound population across SCINS classes for both databases were highly skewed, with most compounds being described by a small number of SCINS classes, and this being more pronounced in ChEMBL.

When benchmarked against Ward’s clustering^9^ in partitioning a set of bioactive molecules for DRD2, SCINS was found to perform more poorly when the goal was to group compounds of similar bioactivity together. However, visual inspection of some of Ward’s clusters and SCINS classes revealed that while a common substructure was identified in each cluster by Ward’s method, this was not a guarantee for overall structural similarity, which would consider size or molecular weight, for example, in agreement with previous findings.^41^ In contrast, SCINS does not contain atom-type content and topology information, but in many cases groups compounds of similar sizes together. Finally, we highlight cases where SCINS is not expected to produce good results, such as when no scaffold is present in the molecule or when there are no chains between rings in a molecule.

On a practical note, we think that SCINS can be a useful tool for the analysis and comparison of large chemical spaces in addition to Murcko and generic Murcko scaffolds because it is fast and more abstracted hence in combination the three methods provide different and complementary insights as illustrated by the ChEMBL and Enamine examples. However, when it comes to grouping bioactive compounds fingerprints should still be considered the go-to method as they capture chemical motifs important for activity. That said, in cases where it is desirable to have a more abstracted scaffold-based view of bioactive compounds sets that generally groups together compounds of similar sizes, then SCINS is an option to consider.

Overall, we hope that this open-source version of SCINS would be an additional tool in the hands of the community in tackling problems associated with the analysis of large databases.

## Data Availability

The code for SCINS is open sourced on: https://github.com/PangeAI/SCINS under an MIT license. ChEMBL v. 33 is available at: https://chembl.gitbook.io/chembl-interface-documentation/downloads. Enamine REAL Diverse is available at: https://enamine.net/compound-collections/real-compounds/real-database-subsets.

## Supporting Information Available

Examples of Murcko scaffolds in Enamine REAL Diverse with SCINS 2_2_3_3_0–3_0_0_0–1_3_0_0 and the same generic Murcko scaffold (Figure S1–Sn); examples of generic Murcko scaffolds in Enamine REAL Diverse with SCINS 2_2_3_3_0–3_0_0_0–1_3_0_0 (Figure S2–Sn); distribution of the number of members per SCINS class in ChEMBL (Figure S3–Sn); distribution of the number of members per SCINS class in Enamine REAL Diverse (Figure S4–Sn); molecular weight distribution of compounds in Enamine REAL Diverse (Figure S5–Sn); molecular weight distribution of small molecules in ChEMBL (Figure S6–Sn); distribution of the number of members per SCINS class in ChEMBL when physicochemical property filters as in obtaining Enamine REAL Diverse are applied (Figure S7–Sn); SCINS retrieval curves for Enamine REAL Diverse and ChEMBL in terms of absolute number of SCINS classes (Figure S8–Sn) (PDF)
