# Supplementary material for: An Open-Source Implementation of the Scaffold Identification and Naming System (SCINS) and Example Applications
Source: J Chem Inf Model. 2024 Oct 15;64(20):7905–16. doi: 10.1021/acs.jcim.4c01314 (PMC11523071; doi:10.1021/acs.jcim.4c01314)
Supplement: Supplementary file 1 — ci4c01314_si_001.pdf [file ci4c01314_si_001.pdf]

Supporting Information: An Open-Source Implementation of the Scaffold Identification and Naming System (SCINS) and Example Applications

Kamen P. Petrov[a], *Andreas Bender*[a,b,*]

[a] Pangea Bio, Pangea Botanica GmbH, Hardenbergstrasse 32, 10623 Berlin, Germany

[b] Centre for Molecular Informatics, Yusuf Hamied Department of Chemistry, University of Cambridge, Lensfield Rd, CB2 1EW, Cambridge, United Kingdom

[*] Email: andreas@pangeabio.com

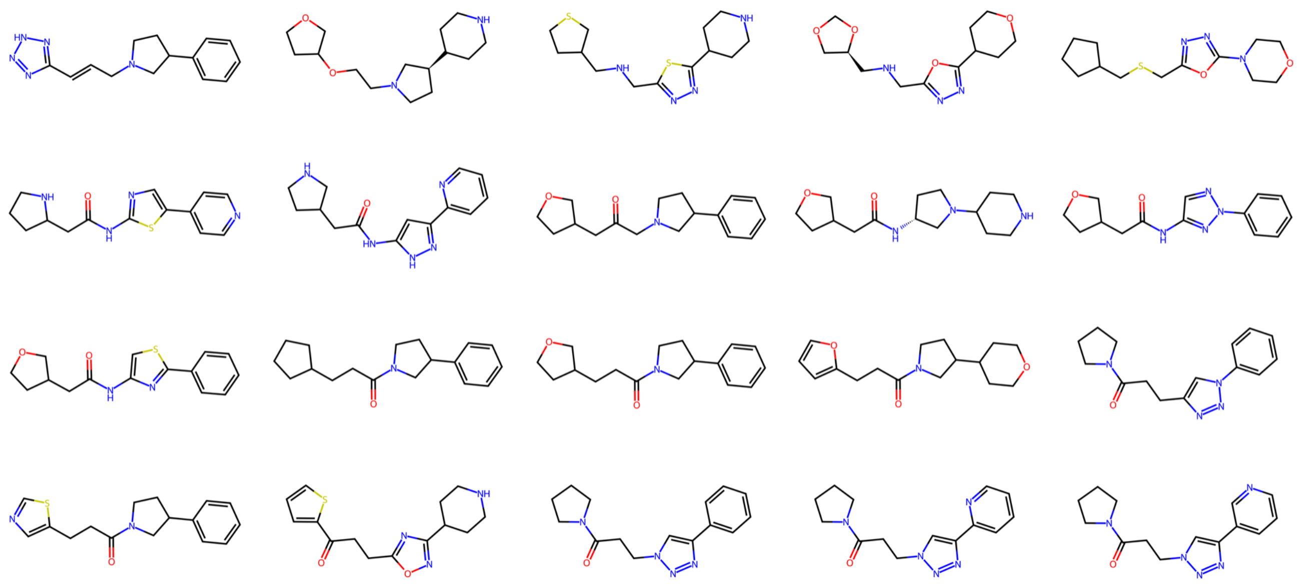

Figure S1-Sn: Examples of Murcko scaffolds in Enamine REAL Diverse with SCINS 2_2_3_3_0-3_0_0_0-1_3_0_0 and the same generic Murcko scaffold (top left in Supplementary Figure 7). The different atom and bond types do not affect the generic Murcko scaffold. Additionally, the position of the carbonyl group (where present) is also disregarded because of the way the generic scaffold was obtained (see Methods).

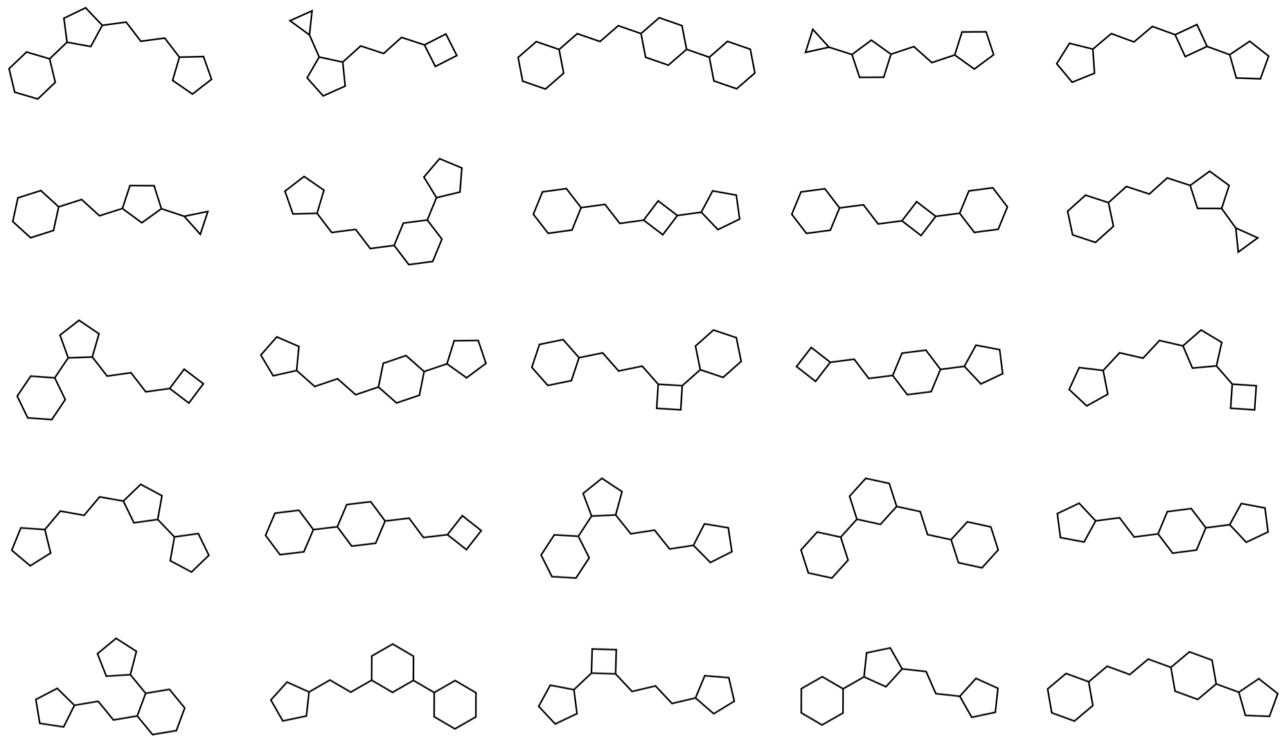

Figure S2-Sn: Examples of generic Murcko scaffolds in Enamine REAL Diverse with SCINS 2_2_3_3_0-3_0_0_0-1_3_0_0. Despite the different topologies and ring sizes SCINS does not change.

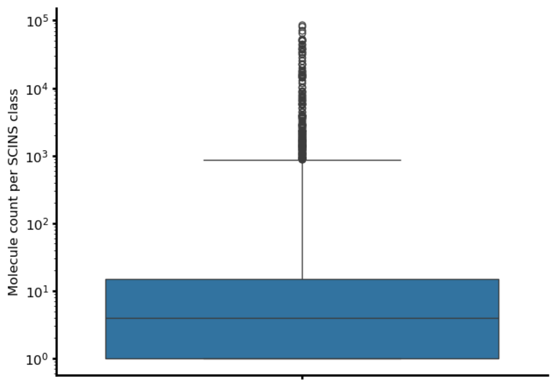

Figure S3-Sn: Distribution of the number of members per SCINS class in ChEMBL.

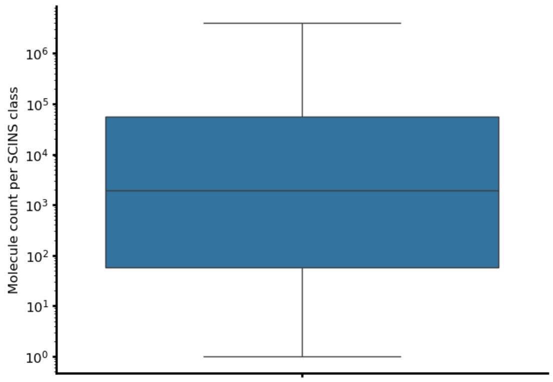

Figure S4-Sn: Distribution of the number of members per SCINS class in Enamine REAL Diverse.

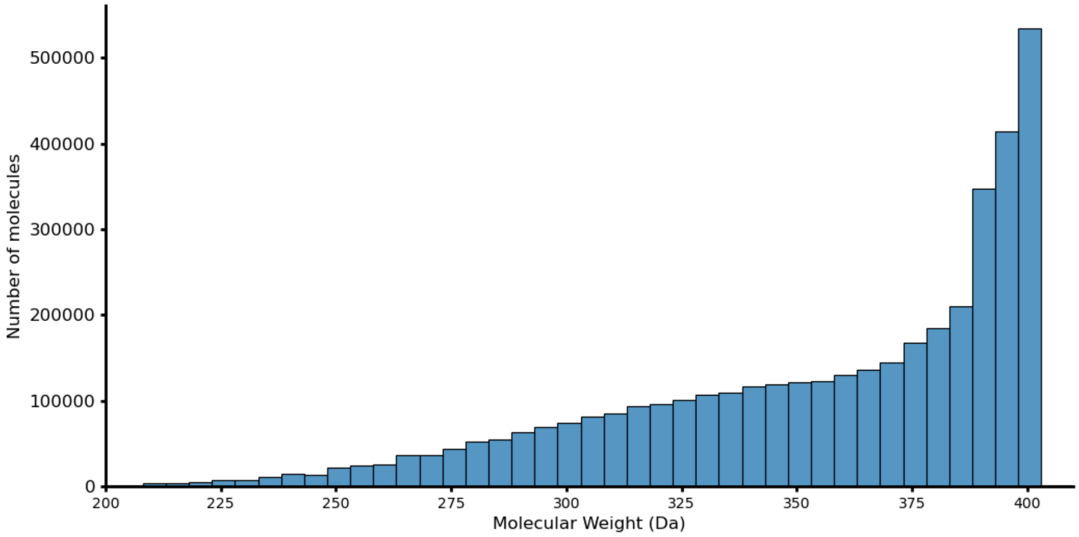

Figure S5-Sn: Molecular weight distribution of 4,000,000 random compounds in Enamine REAL Diverse. X-axis shows only the distribution between 200-410 Da, while less than 1% of the molecular weights are outside this range.

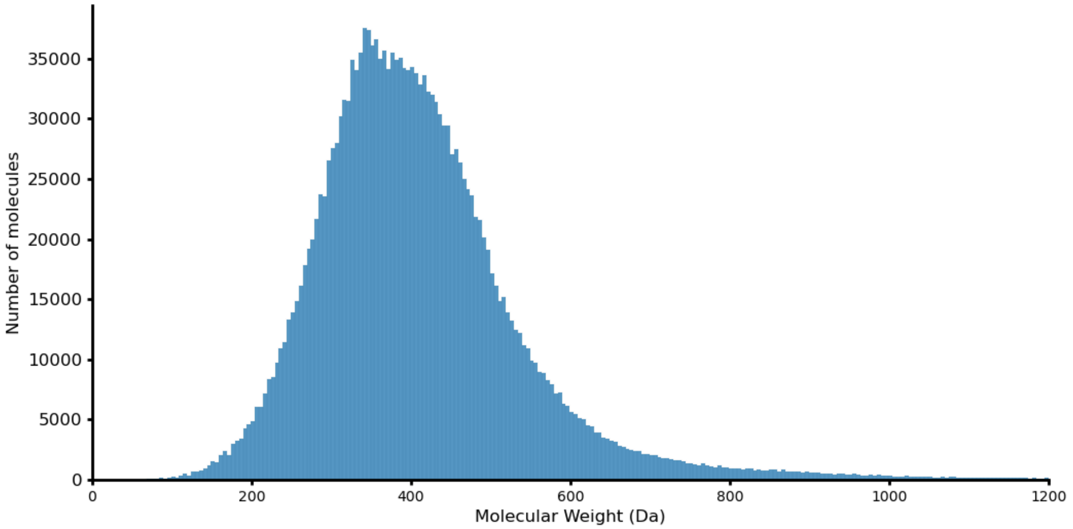

Figure S6-Sn: Molecular weight distribution of small molecules in ChEMBL. The x-axis is cut off at 1200 Da while less than 1% of the molecular weights are above this.

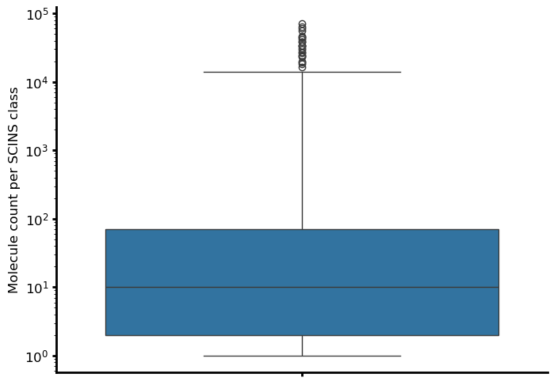

Figure S7-Sn: Distribution of the number of members per SCINS class in ChEMBL when physicochemical property filters as in obtaining Enamine REAL Diverse are applied.

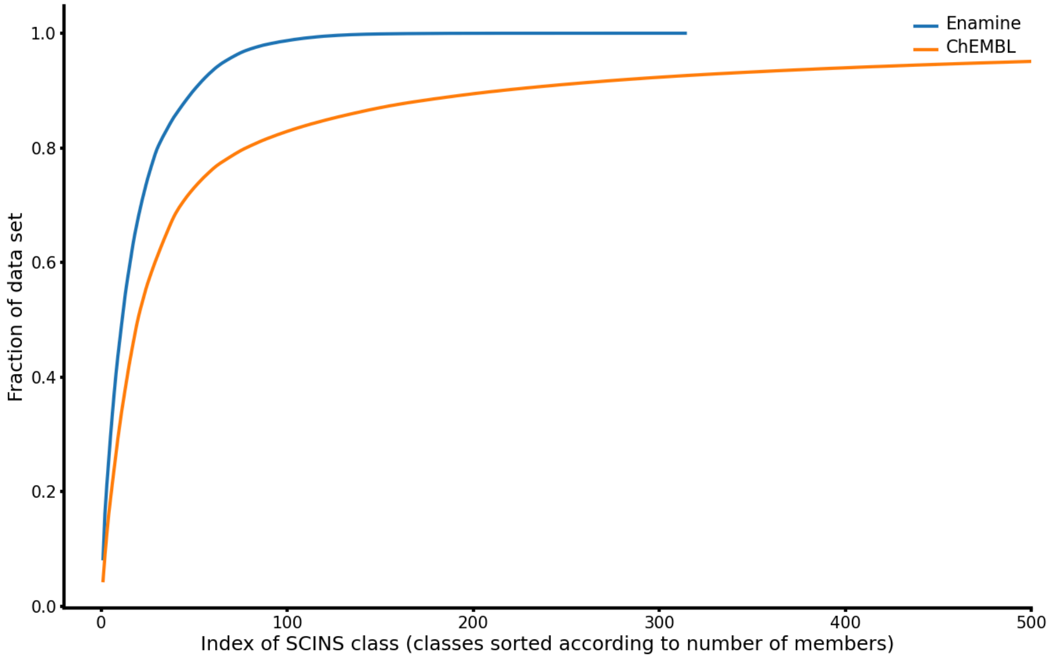

Figure S8-Sn: SCINS retrieval curves for Enamine REAL Diverse and ChEMBL in terms of absolute number of SCINS classes. The x-axis is truncated to 500.
